# Genomic landscape of hyperleukocytic acute myeloid leukemia

**DOI:** 10.1038/s41408-021-00601-5

**Published:** 2022-01-05

**Authors:** Laetitia Largeaud, Sarah Bertoli, Emilie Bérard, Suzanne Tavitian, Muriel Picard, Stéphanie Dufrechou, Naïs Prade, François Vergez, Jean Baptiste Rieu, Isabelle Luquet, Audrey Sarry, Françoise Huguet, Jean Ruiz, Véronique De Mas, Eric Delabesse, Christian Récher

**Affiliations:** 1 Centre Hospitalier Universitaire de Toulouse, Institut Universitaire du Cancer de Toulouse Oncopole, Laboratoire d’Hématologie, Toulouse, France; 2 Université Toulouse III Paul Sabatier, Toulouse, France; 3 Cancer Research Center of Toulouse, UMR1037-INSERM, ERL5294 CNRS Toulouse, France; 4 Centre Hospitalier Universitaire de Toulouse, Institut Universitaire du Cancer de Toulouse Oncopole, Service d’Hématologie, Toulouse, France; 5 Centre Hospitalier Universitaire de Toulouse, Service d’Epidémiologie, Toulouse, France; 6 UMR 1295 CERPOP, INSERM-Université de Toulouse III, Toulouse, France; 7 Service de Réanimation Polyvalente, Centre Hospitalier Universitaire de Toulouse, Institut Universitaire du Cancer de Toulouse Oncopole, Toulouse, France

**Keywords:** Cancer genomics, Acute myeloid leukaemia

**Dear Editor**,

Approximately 20% of patients with acute myeloid leukemia (AML) present at diagnosis with hyperleukocytosis, which is commonly defined as a white blood cell count > 50 ×10^9^/L or > 100 ×10^9^/L [1]. Hyperleukocytic AML is an oncological emergency because the risk of early death is significant due to leukemic organ infiltration, leukostasis syndrome, disseminated intravascular coagulopathy and tumour lysis syndrome. Early management of these symptoms as well as rapid leukoreduction are critical in the therapeutic management [2].

In the era of next-generation sequencing (NGS), considerable progress has been made in understanding the genetic diversity of AML [3–5]. However, owing to the small proportion of hyperleukocytic patients generally included in clinical trials, the genomic landscape of hyperleukocytic AML and the prognostic impact of genetic lesions in this specific clinical context have not been described in detail except in a recent study from Taiwan which reported the frequency of mutations in a panel of 20 myeloid genes [6].

We recently reported the impact of dexamethasone in a series of 160 hyperleukocytic patients [7]. Here, we used this patient series to provide a molecular description of hyperleukocytic AML and to assess the prognostic impact of genetic classifications in patients treated with or without dexamethasone.

DEXAML-00 was a retrospective, single centre study comparing hyperleukocytic AML patients (18–75 years) who received intensive chemotherapy with (*n* = 60) or without (*n* = 100) dexamethasone between 2004 and 2015 [7]. Diagnostic samples for NGS analyses were available for 154 patients (96.3%), 59 patients who received dexamethasone, and 95 patients who did not. Extended DNA resequencing was performed using an Illumina NextSeq500 and Sureselect (Agilent, Santa Clara, CA) targeted on the complete coding regions of 79 genes commonly mutated in myeloid malignancies (Supplementary data). Data were processed using two GATK algorithms, HaplotypeCaller (scaling accurate genetic variant discovery to tens of thousands of samples) and Mutect2, and via Agilent Surecall software, with a sensitivity of 1% [8, 9]. All variants called by two variant callers, were checked using IGV software. Statistical analyses were performed using STATA software 14.2 (STATA Corp., College Station, TX).

Patient characteristics, results and outcome were unchanged compared to the first study (Table S1) [7]. Gene mutation frequency is shown in Fig. 1 and Table S2. The cytogenetic risk was favorable, intermediate or adverse in 15 (9.7%), 121 (78.6%) and 18 (11.7%) patients, respectively. Genetic classifications, including the 2018 genomic classification, ELN 2017 risk classifier, *NPM1*/*FLT3*-ITD/*DNMT3A* mutational status and the functional gene categories, are shown in Table 1[3–5]. A total of 616 mutations were identified with an average of 4 mutations per patient (0–10 mutations/patient). Only one patient with inv(16) had no mutation. *FLT3 (62.3%), NPM1 (52.6%), DNMT3A (34.4%), TET2 (23.4%), NRAS (20.8%)* were the most frequently mutated genes. Of the 71 patients (46%) with *FLT3*-ITD mutations, 32 (45.1%) had an allelic ratio > 0.5. Mutations in the RAS pathway were detected in 67 patients (43.5%), including *NRAS* (20.8%), *PTPN11* (9.7%), *KRAS* (9.1%), and *NF1* (3.9%). Overall, a large majority of patients had mutations in signaling genes (*n* = 131, 85.1%). Drug-actionable mutations such as *FLT3* (*n* = 96), *IDH2* (*n* = 17), *IDH1* (*n* = 14), *KIT* (*n* = 5), *TP53* (*n* = 4), or *JAK2* (*n* = 1) were detected in 113 patients (73.4%). In patients with *FLT3* mutations, 12 had co-mutations in *IDH1*, and 12 patients had co-mutations in *IDH2*. Clinical characteristics and the distribution of actionable mutations according to main molecular subsets of the genomic classification and ELN 2017 are shown in Table S3 and S4.

**Fig. 1:**
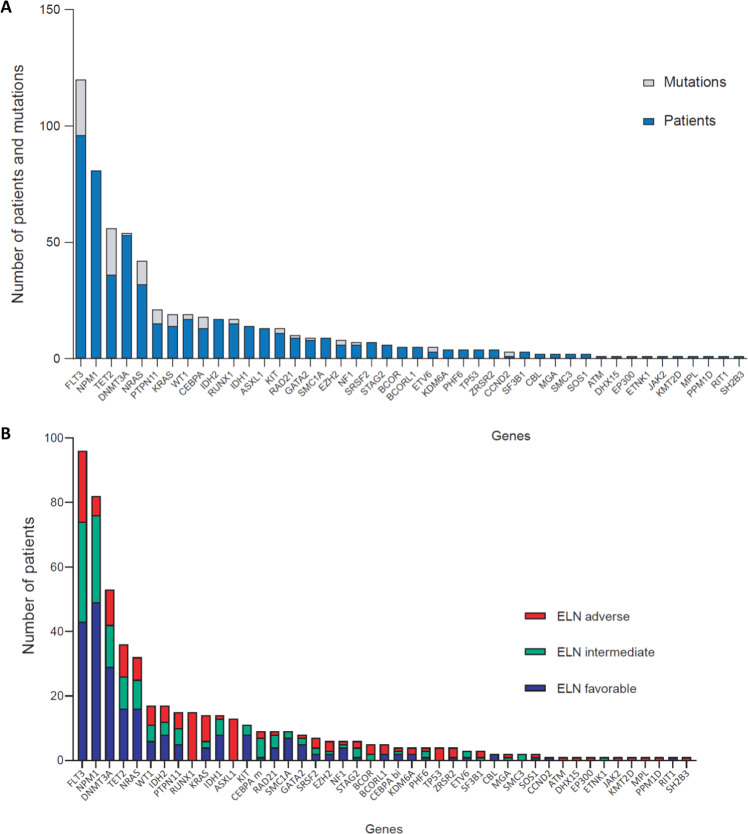
Mutation pattern in hyperleukocytic AML. **A** Number of mutations (grey bars) and patients with mutations (blue bars) per gene. Of the 79 sequenced genes, 44 presented at least one mutation. **B** Distribution of mutations according to the 2017 European Leukaemia Net classification.

**Table 1:** Genetic Classifications of Hyperleukocytic AML.

	Dexamethasone		*P* value	Total 154 (100.0)
	No 95 (%) (61.7)	Yes 59 (%) (38.3)		
Genomic classification* **-** ***n*** **(%)**			0.176	
AML with driver mutations but no detected class-defining lesions	8 (8.4)	8 (13.6)		16 (10.4)
AML with NPM1 mutation	48 (50.5)	32 (54.2)		80 (51.9)
AML with mutated chromatin, RNA-splicing genes, or both	22 (23.2)	7 (11.9)		29 (18.8)
AML with TP53 mutations, chromosomal aneuploidy, or both	4 (4.2)	0 (0.0)		4 (2.6)
AML with inv(16)(p13.1q22) or t(16;16)(p13.1;q22); CBFB–MYH11	6 (6.3)	7 (11.9)		13 (8.4)
AML with biallelic CEBPA mutations	1 (1.1)	2 (3.4)		3 (1.9)
AML with t(8;21)(q22;q22); RUNX1–RUNX1T1	2 (2.1)	0 (0.0)		2 (1.3)
AML with MLL fusion genes; t(x;11)(x;q23)	4 (4.2)	2 (3.4)		6 (3.9)
AML with IDH2R172 mutations and no other class-defining lesions	0 (0.0)	1 (1.7)		1 (0.6)
**ELN 2017** - ***n*** **(%)**			0.231	
Favorable	36 (37.9)	30 (50.8)		66 (42.9)
Intermediate	31 (32.6)	13 (22.0)		44 (28.6)
Adverse	28 (29.5)	16 (27.1)		44 (28.6)
**NPM1/FLT3-ITD/DNMT3A -** ***n*** **(%)**			0.308	
NPM1 = 1, FLT3-ITD = 1, DNMT3A = 1	18 (18.9)	7 (11.9)		25 (16.2)
NPM1 = 1, FLT3-ITD = 1, DNMT3A = 0	10 (10.5)	14 (23.7)		24 (15.6)
NPM1 = 1, FLT3-ITD = 0, DNMT3A = 1	12 (12.6)	6 (10.2)		18 (11.7)
NPM1 = 1, FLT3-ITD = 0, DNMT3A = 0	9 (9.5)	6 (10.2)		15 (9.7)
NPM1 = 0, FLT3-ITD = 1, DNMT3A = 1	5 (5.3)	1 (1.7)		6 (3.9)
NPM1 = 0, FLT3-ITD = 1, DNMT3A = 0	8 (8.4)	8 (13.6)		16 (10.4)
NPM1 = 0, FLT3-ITD = 0, DNMT3A = 1	2 (2.1)	2 (3.4)		4 (2.6)
NPM1 = 0, FLT3-ITD = 0, DNMT3A = 0	31 (32.6)	15 (25.4)		46 (29.9)
**Functional categories -** ***n*** **(%)**				
NPM1 mutation				
No	46 (48.4)	26 (44.1)		72 (46.8)
Yes	49 (51.6)	33 (55.9)	0.598	82 (53.2)
Tumor-suppressor genes				
No	82 (86.3)	47 (79.7)		129 (83.8)
Yes	13 (13.7)	12 (20.3)	0.276	25 (16.2)
DNA methylation-associated genes				
No	36 (37.9)	28 (47.5)		64 (41.6)
Yes	59 (62.1)	31 (52.5)	0.241	90 (58.4)
Signaling genes				
No	14 (14.7)	9 (15.3)		23 (14.9)
Yes	81 (85.3)	50 (84.7)	0.930	131 (85.1)
Myeloid TF gene fusions or mutations				
No	64 (67.4)	37 (62.7)		101 (65.6)
Yes	31 (32.6)	22 (37.3)	0.554	53 (34.4)
Chromatin-modifying genes				
No	77 (81.1)	50 (84.7)		127 (82.5)
Yes	18 (18.9)	9 (15.3)	0.557	27 (17.5)
Cohesin-complex genes				
No	77 (81.1)	50 (84.7)		127 (82.5)
Yes	18 (18.9)	9 (15.3)	0.557	27 (17.5)
Spliceosome-complex genes				
No	85 (89.5)	54 (91.5)		139 (90.3)
Yes	10 (10.5)	5 (8.5)	0.676	15 (9.7)
Others				
No	88 (92.6)	57 (96.6)		145 (94.2)
Yes	7 (7.4)	2 (3.4)	0.483	9 (5.8)

*Compared to the genomic classification, AML with t(15;17)(q22;q12); PML–RARA were excluded, whereas some subsets including AML with inv(3)(q21q26.2) or t(3;3)(q21;q26.2); GATA2, MECOM(EVI1), AML with t(6;9)(p23;q34); DEK–NUP214, AML with no detected driver mutations, AML meeting criteria for ≥2 genomic subgroups were not found in hyperleukocytic AML.

ELN, European Leukemia Net.

We evaluated the prognostic impact of the genetic classifications as well as that of each individual gene according to the treatment group. In all multivariate survival analyses, no significant interaction between dexamethasone treatment and classifications or gene mutations was found, indicating that the effect of dexamethasone did not differ significantly between the various genetic subsets.

When the genomic classification was tested in multivariate analysis, AML with inv(16)/CBFB–MYH11 was the only subgroup with a significant impact on overall survival (OS) (HR, 0.16; 95%CI: 0.03–0.75; *P* = 0.020).

The co-occurrence of *NPM1*/*FLT3*-ITD/*DNMT3A* triple mutations has been shown to be associated with very poor OS.[5] This mutational status was observed in 25 patients (16%), 23 of whom died. Compared to this triple mutated subset, lower HRs were found in double mutant *NPM1*mut/*FLT3*-ITD (HR, 0.43; 95% CI: 0.19–0.97; *P* = 0.041) or *NPM1*mut/*DNMT3A*mut (HR, 0.47; 95% CI: 0.21–1.07; *P* = 0.074).

Regarding functional gene categories, 2 subsets (*NPM1*mut: HR, 0.56; 95% CI: 0.33-0.97; *P* = 0.039 and myeloid transcription factor gene fusions or mutations: HR, 0.34; 95% CI: 0.19–0.60; *P* < 0.001) were significantly and independently associated with better OS whereas the chromatin-modifying gene subset was associated with poorer OS (HR, 1.88; 95% CI: 1.04–3.41; *P* = 0.037).

The ELN 2017 adverse group was independently associated with poor OS (HR, 2.53; 95% CI: 1.46–4.41; *P* = 0.001). However, there was no significant difference between intermediate and favorable prognostic groups (HR, 1.47; 95% CI: 0.87–2.48; *P* = 0.148).

Finally, we assessed the prognostic impact of each individual gene using the least absolute shrinkage and selection operator (LASSO) statistical method (Table S5). *DNMT3A* mutations were independently predictive of poor OS (HR, 1.76; 95% CI: 1.02–3.03; *P* = 0.043). On the contrary, *CBFB-MYH11* (HR, 0.10; 95% CI: 0.02–0.43; *P* = 0.002), *CEBPA* (HR, 0.22; 95% CI: 0.09–0.53; *P* = 0.001), *NPM1* (HR, 0.33; 95% CI: 0.19–0.58; *P* < 0.001) and surprisingly, *RUNX1* mutations (HR, 0.40; 95% CI: 0.18–0.92; *P* = 0.030) were significantly and independently associated with better OS. The different types of *RUNX1* mutations, co-mutations, response to treatment with or without dexamethasone and outcome are shown in Table S6. Of the 15 patients with *RUNX1*^mut^ AML, 13 (86.7%) achieved a complete response.

Multivariate analyses for event-free survival, relapse-free survival and cumulative incidence of relapse yielded similar results (data not shown). Of note, clinical or treatment parameters including infection at diagnosis, secondary AML, hydroxyurea, albumin, LDH, fibrinogen, CD56 expression, admission to intensive care unit (ICU) or allogeneic stem-cell transplantation retained an independent prognostic value in most multivariate analyses (Table S5).

This study shows that the genomics of hyperleukocytic AML differs substantially from nonhyperleukocytic AML. Signalling mutations in either *FLT3* or RAS pathways, mutations in DNA methylation genes and *NPM1* were over-represented whereas other subgroups, such as *RUNX1-RUNX1T1* or *TP53* mutations had a very low frequency. Compared to the Taiwanese study, we found far fewer *CEBPA* biallelic mutations (2.6% vs. 16%) and more *NPM1* (53.2% vs. 30%) or *FLT3* mutations (62.3% vs. 44.5%). Apart from the difference in median age between the two cohorts (60 vs. 50 y), we have no clear explanation for this difference [6].

A large proportion of hyperleukocytic AML patients present at diagnosis with therapeutically targetable mutations (>70%). This brings hope that the use of targeted therapies may improve their prognosis, which is still poor compared to nonhyperleukocytic AML. Midostaurin combined with intensive chemotherapy has improved the outcome of AML patients with *FTL3* mutations (~60% of patients in our series) including ELN 2017 adverse risk patients [10]. Our patients were treated before midostaurin approval in Europe and therefore no patient received a *FLT3* inhibitor. Thus, further studies are needed to assess the impact of midostaurin in hyperleukocytic AML. Inhibition of the RAS pathway could also be a valuable avenue in this setting [11].

AML with *RUNX1* mutations (*RUNX1*^mut^ AML) is a provisional entity of the WHO 2016 classification that accounts for 10% of newly diagnosed AML. In our series of hyperleukocytic patients, the frequency of *RUNX1* mutations was similar (*n* = 15, 9.7%). *RUNX1*^mut^ AML are classified in the ELN 2017 adverse risk group. However, *RUNX1* mutations were independently associated with better OS in our cohort. A recent study using a chemogenomic approach revealed that AML cells with inactivating *RUNX1* mutations were particularly sensitive to very low concentrations of glucocorticoids [12]. However, given the small patient cohort, we were unable to link this improved outcome to dexamethasone treatment—hence the clinical impact of glucocorticoids in *RUNX1*^mut^ AML has yet to be determined.

Studies on the mechanisms of resistance to chemotherapy or tyrosine kinase inhibitors together with high-throughput drug screening have underpinned the potential role of glucocorticoids in AML. Cytarabine resistance is associated with the acquisition of sensitivity to glucocorticoids and mutated *RUNX1*, *NPM1*, or *SRSF2* modulate gene expression in a manner that primes AML cells for glucocorticoid sensitivity [13]. These recent data suggest that some AML subgroups (not necessarily hyperleukocytic patients) may specifically benefit from dexamethasone. However, we did not find any significant interaction between dexamethasone treatment and genomic alterations. This may be due to insufficient numbers or dexamethasone may have broader effects on biological phenomena such as inflammation, microenvironment or leukemic stem-cell biology.

Hyperleukocytic AML remains a very challenging clinical management issue. This is evidenced by the high number of multivariate analysis parameters that reflect the patients’ general condition, inflammation, metabolism or therapeutic management. Acting on these parameters could be key to limiting early complications and induction deaths. Leukoreduction with hydroxyurea, anti-inflammatory treatments, such as dexamethasone and early or direct admission to ICU have recently been proposed to this end whereas the benefit of leukapheresis has not been demonstrated [14]. New targeted therapies that may cover the majority of hyperleukocytic patients as shown in our study and a better understanding of the mechanisms of leukostasis could help to improve patient management and prognosis [15].

## Supplementary information

Supplemental Material
