# Supplementary material for: Genomic landscape of hyperleukocytic acute myeloid leukemia
Source: Blood Cancer J. 2022 Jan 5;12(1):4. doi: 10.1038/s41408-021-00601-5 (PMC8733030; doi:10.1038/s41408-021-00601-5)
Supplement: Supplementary file 1 — Supplemental Material [file 41408_2021_601_MOESM1_ESM.docx]

**Supplementary data**

**Genomic landscape of hyperleukocytic acute myeloid leukemia**

Laetitia Largeaud et al.

**Subjects and Methods**

**Patients**

The study population comprised 160 patients (18 – 75 years old) with hyperleukocytic AML (WBC≥ 50 giga/L) receiving intensive chemotherapy between 2004 and 2015. In this pre-midostaurin registration patient cohort, no patient received a *FLT3* inhibitor. Endpoint criteria and outcomes have been previously reported (Bertoli S, Haematologica. 2018 Jun; 103(6):988-98). In this earlier study, multivariate analysis showed that dexamethasone was significantly associated with improved relapse incidence, disease-free survival (DFS), event-free survival (EFS) and overall survival (OS). Diagnostic samples for NGS analyses were available for 154 patients (96.3% of the initial cohort). Further details regarding the patient population and treatments are given in the primary publication. This study was performed in accordance with the Declaration of Helsinki. All registries were approved by institutional review boards or national authorities, and informed consent was obtained from all patients.

**Next-generation sequencing (gene panel)**

ADN samples were collected from AML patients, after obtaining informed consent, and stored in the HIMIP collection (BB-0033-00060). In accordance with French legislation, HIMIP collections have been declared to the Ministry of Higher Education and Research (DC 2008-307 collection 1) and issued with a transfer agreement (AC 2008-129) following approval by the “Comité de Protection des Personnes Sud-Ouest et Outremer II” (South West and Overseas II Ethics Committee). Extended DNA resequencing was performed using an Illumina NextSeq500 and Sureselect (Agilent, Santa Clara, CA) targeted on the complete coding regions of 90 genes commonly mutated in myeloid malignancies: *ACD (NM_001082486.2); ADGRV1 (NM_032119.4); ANKRD26 (NM_014915.3); ASXL1 (NM_015338.5); ASXL2 (NM_018263.6); ATM (NM_000051.3); BCOR (NM_017745.5); BCORL1 (NM_021946.4); BRAF (NM_004333.6); CALR (NM_004343.4); CBL (NM_005188.3); CCND2 (NM_001759.4); CEBPA (NM_004364.4); CSF3R (NM_156039.3); CSMD1 (NM_033225.6); CUX1 (NM_181552.4); DDX41 (NM_016222.4); DHX15 (NM_001358.3); DNMT3A (NM_022552.4); ELANE (NM_001972.4); EP300 (NM_001429.3); ERCC6L2 (NM_020207.7); ETNK1 (NM_018638.4); ETV6 (NM_001987.4); EZH2 (NM_004456.4); FANCA (NM_000135.4); FLT3 (NM_004119.2); GATA1 (NM_002049.4); GATA2 (NM_032638.4); HAX1 (NM_006118.4); IDH1 (NM_005896.3); IDH2 (NM_002168.3); JAK2 (NM_004972.3); JAK3 (NM_000215.4); KDM5A (NM_001042603.3); KDM6A (NM_021140.3); KIT (NM_000222.2); KMT2D (NM_003482.3); KRAS (NM_004985.4); MECOM (NM_001105077.4); MGA (NM_001164273.1); MPL (NM_005373.2); MYC (NM_002467.6); NF1 (NM_000267.3); NPM1 (NM_002520.6); NRAS (NM_002524.4); PHF6 (NM_032335.3); PIGA (NM_002641.4); PPM1D (NM_003620.3); PRPF8 (NM_006445.4); PTPN11 (NM_002834.3); RAD21 (NM_006265.2); RIT1 (NM_006912.5); RRAS (NM_006270.5); RTEL1 (NM_001283009.2); RUNX1 (NM_001754.4); SAMD9 (NM_017654.4); SAMD9L (NM_152703.5); SBDS (NM_016038.4); SETBP1 (NM_015559.3); SF3B1 (NM_012433.3); SH2B3 (NM_005475.2); SLC37A4 (NM_001164277.2); SMC1A (NM_006306.3); SMC3 (NM_005445.3); SOS1 (NM_005633.3); SRP72 (NM_006947.4); SRSF2 (NM_003016.4); STAG2 (NM_006603.4); TERC ; TERT (NM_198253.3); TET2 (NM_001127208.2); TP53 (NM_001126112.2); U2AF1 (NM_006758.3); USP7 (NM_003470.3); WAS (NM_000377.3); WT1 (NM_000378.4); ZBTB7A (NM_015898.4); ZRSR2 (NM_005089.3).* Data were processed using two GATK algorithms (<https://software.broadinstitute.org/gatk>), HaplotypeCaller (scaling accurate genetic variant discovery to tens of thousands of samples) and Mutect2, and also via Agilent Surecall software, with a sensitivity of 1% (McKenna A, Genome Res. 2010 Sep;20(9):1297-303; Poplin R, bioRxiv. 2018:201178). All variants called by two variant callers, were checked using IGV software. Identified variants were curated manually and named as per the rules of the Human Genome Variation Society ([hgvs.org](http://hgvs.org/)). The presence of *FLT3*-ITD was tested as described (LaRochelle O, Oncotarget. 2011 Nov;2(11):850-61). Electrophoregram peaks were quantified using GeneMarker 2.2 (SoftGenetics, State College, PA). *CEBPA* screening was performed by classical Sanger sequencing according to Pabst *et al*. (Pabst T, Nat Genet. 2001 Mar;27(3):263-70).

**Statistical analyses**

Statistical analyses were performed using STATA software 14.2 (STATA Corp., College Station, TX, USA). All reported P-values were two-sided and the significance threshold was set at <0.05. For survival end points (OS, EFS, DFS), hazard ratio (HR) and 95% confidence intervals (CI) were assessed using a standard Cox model. A proportional sub-distribution hazard model (which is an extension of the Cox model for competing risk situations) was used for the cumulative incidence of relapse (CIR) (Fine JP, Gray RJ, Journal of the American Statistical Association. 1999;94(446):496-509). The variables initially introduced into the multivariate survival analyses, together with dexamethasone and each genetic classification (the genomic classification, *NPM1*/*FLT3*-ITD/*DNMT3A* mutational status, functional gene categories and ELN2017), were all risk factors (potential confounding factors) identified in the primary publication (Bertoli S, Haematologica. 2018 Jun; 103(6):988-98). Backward analysis was then applied. The proportional hazard assumption was tested for each covariate and was always met. Interactions between each genetic classification and dexamethasone were tested. Allogeneic stem-cell transplantation was evaluated as a time-dependent covariate. As the correlation between each individual gene mutation was very high (multicollinearity), the traditional multivariate Cox model produces estimates that are both biased and unstable, leading to an arbitrary selection of important variables. The penalised regression methods developed to overcome this statistical problem include L1 Penalised Cox regression using LASSO (least absolute shrinkage and selection operator) (Tibshirani R. Stat Med. 1997 Feb 28;16(4):385-95). LASSO tends to select only a few variables from a set of highly correlated variables, thereby facilitating variable selection and the selection of a sparser model. The regularisation parameter used for penalisation was estimated using 10-fold cross validation and applied to the mutations. This analysis was repeated on 500 replications obtained by bootstrapping the original data (BOLASSO) in order to produce a consistent selection of mutations. The mutations selected after bootstrapping were the only non-zero mutations (i.e. having a predictive value) in at least 80% of the replications. Once the mutations were selected, a Cox model including dexamethasone and the selected mutations was fitted with further adjustment for previously known confounding factors (Bertoli S, Haematologica. 2018). We then proceeded as described above (for the impact of each genetic classification) using backward analysis and testing interactions between selected mutations and dexamethasone.

**Supplementary Table 1: Patient Characteristics and outcome**

|  | **Dexamethasone** | |  |  |
| --- | --- | --- | --- | --- |
|  | **No** | **Yes** | ***P*** | **Total** |
|  | 95 (61.7) | 59 (38.3) |  | 154 (100.0) |
| **Gender - n (%)** |  |  | .278 |  |
| Male | 52 (54.7) | 27 (45.8) |  | 79 (51.3) |
| Female | 43 (45.3) | 32 (54.2) |  | 75 (48.7) |
| **Age - years** |  |  |  |  |
| n /missing | 95/0 | 59/0 |  | 154/0 |
| Mean (SD) | 57.62 (13.26) | 55.62 (15.15) | .389 | 56.85 (14.00) |
| Median | 60.62 | 59.24 |  | 59.83 |
| IQR | [47.70; 68.67] | [ 51.24; 66.58] |  | [ 48.69; 67.20] |
| Min; Max | 20.79; 75.88 | 18.64; 75.53 |  | 18.64; 75.88 |
| **Age - n (%)** |  |  | .482 |  |
| <60 | 46 (48.4) | 32 (54.2) |  | 78 (50.6) |
| >=60 | 49 (51.6) | 27 (45.8) |  | 76 (49.4) |
| **ECOG performance, status - n (%)** |  |  | .001 |  |
| 0-1 | 59 (81.9) | 30 (55.6) |  | 89 (70.6) |
| 2-3-4 | 13 (18.1) | 24 (44.4) |  | 37 (29.4) |
| **Extramedullary involvement - n (%)** |  |  | .533 |  |
| No | 36 (46.8) | 24 (41.4) |  | 60 (44.4) |
| Yes | 41 (53.2) | 34 (58.6) |  | 75 (55.6) |
| **AML status - n (%)** |  |  | .104 |  |
| De novo | 78 (82.1) | 54 (91.5) |  | 132 (85.7) |
| Secondary | 17 (17.9) | 5 (8.5) |  | 22 (14.3) |
| **Leukostasis - n (%)** |  |  | .000* |  |
| No | 81 (85.3) | 33 (55.9) |  | 114 (74.0) |
| CNS | 2 (2.1) | 9 (15.3) |  | 11 (7.1) |
| Lung | 8 (8.4) | 12 (20.3) |  | 20 (13.0) |
| CNS and lung | 4 (4.2) | 5 (8.5) |  | 9 (5.8) |
| **Infection at diagnosis – n (%)** |  |  | .541 |  |
| No | 74 (80.4) | 45 (76.3) |  | 119 (78.8) |
| Yes | 18 (19.6) | 14 (23.7) |  | 32 (21.2) |
| **White cell count - x 10^9^/L** |  |  |  |  |
| n /missing | 95/0 | 59/0 |  | 154/0 |
| Mean (SD) | 105.05 (57.67) | 138.42 (68.26) | .001 | 117.84 (63.83) |
| Median | 85.80 | 118.92 |  | 97.60 |
| IQR | [65.80; 113.10] | [90.40; 180.90] |  | [70.80; 137.00] |
| Min; Max | 50.40; 327.00 | 50.66; 42.00 |  | 50.40; 342.00 |
| **White cell count – x 10^9^/L – n (%)** |  |  | .001 |  |
| <100 | 59 (62.1) | 21 (35.6) |  | 80 (51.9) |
| >=100 | 36 (37.9) | 38 (64.4) |  | 74 (48.1) |
| **Platelet count - x 10^9^/L - n (%)** |  |  | .549 |  |
| <50 | 42 (44.2) | 29 (49.2) |  | 71 (46.1) |
| >=50 | 53 (55.8) | 30 (50.8) |  | 83 (53.9) |
| **Cytogenetic risk – n (%)** |  |  | .521 |  |
| Favorable | 8 (8.4) | 7 (11.9) |  | 15 (9.7) |
| Intermediate | 74 (77.9) | 47 (79.7) |  | 121 (78.6) |
| Adverse | 13 (13.7) | 5 (8.5) |  | 18 (11.7) |
| **CD56 - n (%)** |  |  | .676 |  |
| ≤20% | 69 (78.4) | 43 (75.4) |  | 112 (77.2) |
| >20% | 19 (21.6) | 14 (24.6) |  | 33 (22.8) |
| **Creatinine (µM/L) - n (%)** |  |  | .880 |  |
| <=120 | 78 (82.1) | 49 (83.1) |  | 127 (82.5) |
| >120 | 17 (17.9) | 10 (16.9) |  | 27 (17.5) |
| **Bilirubin (µM/L) - n (%)** |  |  | 1 |  |
| <=25 | 91 (95.8) | 54 (94.7) |  | 145 (95.4) |
| >25 | 4 (4.2) | 3 (5.3) |  | 7 (4.6) |
| **Albumin (g/L) - n (%)** |  |  | .342 |  |
| <35 | 38 (40.4) | 28 (48.3) |  | 66 (43.4) |
| >=35 | 56 (59.6) | 30 (51.7) |  | 86 (56.6) |
| **Ferritin (µg/L) - n (%)** |  |  | .564 |  |
| <=1000 | 23 (51.1) | 24 (45.3) |  | 47 (48.0) |
| >1000 | 22 (48.9) | 29 (54.7) |  | 51 (52.0) |
| **LDH - n (%)** |  |  | .532 |  |
| <=1550 | 45 (47.4) | 31 (52.5) |  | 76 (49.4) |
| >1550 | 50 (52.6) | 28 (47.5) |  | 78 (50.6) |
| **Fibrinogen (g/L) - n (%)** |  |  | .345 |  |
| <=1.5 | 7 (7.4) | 7 (11.9) |  | 14 (9.1) |
| >1.5 | 88 (92.6) | 52 (88.1) |  | 140 (90.9) |
| **Study period - n (%)** |  |  | <.0001 |  |
| 2004-2009 | 64 (67.4) | 0 (0.0) |  | 64 (41.6) |
| 2010-2015 | 31 (32.6) | 59 (100.0) |  | 90 (58.4) |
| **Hydroxyurea - n (%)** |  |  | .005 |  |
| No | 38 (40.0) | 11 (18.6) |  | 49 (31.8) |
| Yes | 57 (60.0) | 48 (81.4) |  | 105 (68.2) |
| **Admission to intensive care unit - n (%)** |  |  | <.0001 |  |
| No | 80 (84.2) | 31 (52.5) |  | 111 (72.1) |
| Yes | 15 (15.8) | 28 (47.5) |  | 43 (27.9) |
| **Allo-SCT n(%)** |  |  | .477 |  |
| No | 71 (74.7) | 41 (69.5) |  | 112 (72.7) |
| Yes | 24 (25.3) | 18 (30.5) |  | 42 (27.3) |
| **Day-60 deaths - n (%)** |  |  | .190 |  |
| No | 76 (80.0) | 52 (88.1) |  | 128 (83.1) |
| Yes | 19 (20.0) | 7 (11.9) |  | 26 (16.9) |
| **Induction failure - n (%)** |  |  | 1 |  |
| No | 89 (93.7) | 56 (94.9) |  | 145 (94.2) |
| Yes | 6 (6.3) | 3 (5.1) |  | 9 (5.8) |
| **Complete response - n (%)** |  |  | .285 |  |
| No | 23 (24.2) | 10 (16.9) |  | 33 (21.4) |
| Yes | 72 (75.8) | 49 (83.1) |  | 121 (78.6) |
| **Event-free survival - months** |  |  |  |  |
| Median (IQR) | 11.3 (3.5-26.1) | 39.4 (8.0-NR) | .002 |  |
| **Disease-free survival - months** |  |  |  |  |
| Median (IQR) | 13.6 (6.5-NR) | 66.3 (12.7-NR) | .002 |  |
| **Overall survival - months** |  |  |  |  |
| Median (IQR) | 18.3 (5.4-67.0) | NR (10.6-NR) | .006 |  |

IQR, interquartile range; ECOG, Eastern Cooperative Oncology Group; CNS, central nervous system; ELN, European Leukemia Net; DA, daunorubicin-cytarabine; IA, idarubicin-cytarabine; Allo-SCT, allogeneic stem cell transplantation; IQR, interquartile range; NR, not reached.

**Supplementary Table 2: Frequency of Gene Mutations in Hyperleukocytic AML**

|  | **Dexamethasone** | |  |  |
| --- | --- | --- | --- | --- |
|  | **No** | **Yes** | ***P* value** | **Total** |
|  | 95 (61.7) | 59 (38.3) |  | 154 (100.0) |
| RUNX1-RUNX1T1 n(%) |  |  | .524 |  |
| No | 93 (97.9) | 59 (100.0) |  | 152 (98.7) |
| Yes | 2 (2.1) | 0 (0.0) |  | 2 (1.3) |
| CBFB-MYH11 n(%) |  |  | .246 |  |
| No | 89 (93.7) | 52 (88.1) |  | 141 (91.6) |
| Yes | 6 (6.3) | 7 (11.9) |  | 13 (8.4) |
| MLL Fusion n(%) |  |  | .711 |  |
| No | 89 (93.7) | 57 (96.6) |  | 146 (94.8) |
| Yes | 6 (6.3) | 2 (3.4) |  | 8 (5.2) |
| ASXL1 mutation n(%) |  |  | .133 |  |
| No | 84 (88.4) | 57 (96.6) |  | 141 (91.6) |
| Yes | 11 (11.6) | 2 (3.4) |  | 13 (8.4) |
| ASXL2 mutation n(%) |  |  | . |  |
| No | 95 (100.0) | 59 (100.0) |  | 154 (100.0) |
| ATM mutation n(%) |  |  | 1 |  |
| No | 94 (98.9) | 59 (100.0) |  | 153 (99.4) |
| Yes | 1 (1.1) | 0 (0.0) |  | 1 (0.6) |
| BCOR mutation n(%) |  |  | .157 |  |
| No | 90 (94.7) | 59 (100.0) |  | 149 (96.8) |
| Yes | 5 (5.3) | 0 (0.0) |  | 5 (3.2) |
| BCORL1 mutation n(%) |  |  | 1 |  |
| No | 92 (96.8) | 57 (96.6) |  | 149 (96.8) |
| Yes | 3 (3.2) | 2 (3.4) |  | 5 (3.2) |
| CBL mutation n(%) |  |  | .524 |  |
| No | 93 (97.9) | 59 (100.0) |  | 152 (98.7) |
| Yes | 2 (2.1) | 0 (0.0) |  | 2 (1.3) |
| CCND2 mutation n(%) |  |  | 1 |  |
| No | 94 (98.9) | 59 (100.0) |  | 153 (99.4) |
| Yes | 1 (1.1) | 0 (0.0) |  | 1 (0.6) |
| CEBPA mutation n(%) |  |  | .562 |  |
| No | 88 (92.6) | 53 (89.8) |  | 141 (91.6) |
| Yes | 7 (7.4) | 6 (10.2) |  | 13 (8.4) |
| CEBPA monoallelic mutation n(%) |  |  | 1 |  |
| No | 89 (93.7) | 56 (94.9) |  | 145 (94.2) |
| Yes | 6 (6.3) | 3 (5.1) |  | 9 (5.8) |
| CEBPA_biallelic mutation n(%) |  |  | .157 |  |
| No | 94 (98.9) | 56 (94.9) |  | 150 (97.4) |
| Yes | 1 (1.1) | 3 (5.1) |  | 4 (2.6) |
| CSF3R mutation n(%) |  |  | . |  |
| No | 95 (100.0) | 59 (100.0) |  | 154 (100.0) |
| DHX15 mutation n(%) |  |  | 1 |  |
| No | 94 (98.9) | 59 (100.0) |  | 153 (99.4) |
| Yes | 1 (1.1) | 0 (0.0) |  | 1 (0.6) |
| DNMT3A mutation n(%) |  |  | .133 |  |
| No | 58 (61.1) | 43 (72.9) |  | 101 (65.6) |
| Yes | 37 (38.9) | 16 (27.1) |  | 53 (34.4) |
| EP300 mutation n(%) |  |  | .383 |  |
| No | 95 (100.0) | 58 (98.3) |  | 153 (99.4) |
| Yes | 0 (0.0) | 1 (1.7) |  | 1 (0.6) |
| ETV6 mutation n(%) |  |  | .286 |  |
| No | 92 (96.8) | 59 (100.0) |  | 151 (98.1) |
| Yes | 3 (3.2) | 0 (0.0) |  | 3 (1.9) |
| EZH2 mutation n(%) |  |  | 1 |  |
| No | 91 (95.8) | 57 (96.6) |  | 148 (96.1) |
| Yes | 4 (4.2) | 2 (3.4) |  | 6 (3.9) |
| ETNK1 mutation n(%) |  |  | 1 |  |
| No | 94 (98.9) | 59 (100.0) |  | 153 (99.4) |
| Yes | 1 (1.1) | 0 (0.0) |  | 1 (0.6) |
| FLT3-ITD or TKD mutation n(%) |  |  | .447 |  |
| No | 38 (40.0) | 20 (33.9) |  | 58 (37.7) |
| Yes | 57 (60.0) | 39 (66.1) |  | 96 (62.3) |
| FLT3-TKD mutation n(%) |  |  | .836 |  |
| No | 76 (80.0) | 48 (81.4) |  | 124 (80.5) |
| Yes | 19 (20.0) | 11 (18.6) |  | 30 (19.5) |
| GATA1 mutation n(%) |  |  | . |  |
| No | 95 (100.0) | 59 (100.0) |  | 154 (100.0) |
| GATA2 mutation n(%) |  |  | .261 |  |
| No | 92 (96.8) | 54 (91.5) |  | 146 (94.8) |
| Yes | 3 (3.2) | 5 (8.5) |  | 8 (5.2) |
| IDH1 mutation n(%) |  |  | .172 |  |
| No | 84 (88.4) | 56 (94.9) |  | 140 (90.9) |
| Yes | 11 (11.6) | 3 (5.1) |  | 14 (9.1) |
| IDH2 mutation n(%) |  |  | .786 |  |
| No | 84 (88.4) | 53 (89.8) |  | 137 (89.0) |
| Yes | 11 (11.6) | 6 (10.2) |  | 17 (11.0) |
| JAK2 mutation n(%) |  |  | .383 |  |
| No | 95 (100.0) | 58 (98.3) |  | 153 (99.4) |
| Yes | 0 (0.0) | 1 (1.7) |  | 1 (0.6) |
| KDM5A mutation n(%) |  |  | . |  |
| No | 95 (100.0) | 59 (100.0) |  | 154 (100.0) |
| KDM6A mutation n(%) |  |  | .020 |  |
| No | 95 (100.0) | 55 (93.2) |  | 150 (97.4) |
| Yes | 0 (0.0) | 4 (6.8) |  | 4 (2.6) |
| KIT mutation n(%) |  |  | .749 |  |
| No | 89 (93.7) | 54 (91.5) |  | 143 (92.9) |
| Yes | 6 (6.3) | 5 (8.5) |  | 11 (7.1) |
| KMT2D mutation n(%) |  |  | .383 |  |
| No | 95 (100.0) | 58 (98.3) |  | 153 (99.4) |
| Yes | 0 (0.0) | 1 (1.7) |  | 1 (0.6) |
| KRAS mutation n(%) |  |  | .833 |  |
| No | 86 (90.5) | 54 (91.5) |  | 140 (90.9) |
| Yes | 9 (9.5) | 5 (8.5) |  | 14 (9.1) |
| MGA mutation n(%) |  |  | 1 |  |
| No | 94 (98.9) | 58 (98.3) |  | 152 (98.7) |
| Yes | 1 (1.1) | 1 (1.7) |  | 2 (1.3) |
| MPL mutation n(%) |  |  | 1 |  |
| No | 94 (98.9) | 59 (100.0) |  | 153 (99.4) |
| Yes | 1 (1.1) | 0 (0.0) |  | 1 (0.6) |
| MYC mutation n(%) |  |  | . |  |
| No | 95 (100.0) | 59 (100.0) |  | 154 (100.0) |
| NF1 mutation n(%) |  |  | .407 |  |
| No | 90 (94.7) | 58 (98.3) |  | 148 (96.1) |
| Yes | 5 (5.3) | 1 (1.7) |  | 6 (3.9) |
| NPM1 mutation n(%) |  |  | .598 |  |
| No | 46 (48.4) | 26 (44.1) |  | 72 (46.8) |
| Yes | 49 (51.6) | 33 (55.9) |  | 82 (53.2) |
| NRAS mutation n(%) |  |  | .081 |  |
| No | 71 (74.7) | 51 (86.4) |  | 122 (79.2) |
| Yes | 24 (25.3) | 8 (13.6) |  | 32 (20.8) |
| PHF6 mutation n(%) |  |  | 1 |  |
| No | 92 (96.8) | 58 (98.3) |  | 150 (97.4) |
| Yes | 3 (3.2) | 1 (1.7) |  | 4 (2.6) |
| PPM1D mutation n(%) |  |  | 1 |  |
| No | 94 (98.9) | 59 (100.0) |  | 153 (99.4) |
| Yes | 1 (1.1) | 0 (0.0) |  | 1 (0.6) |
| PTPN11 mutation n(%) |  |  | .328 |  |
| No | 84 (88.4) | 55 (93.2) |  | 139 (90.3) |
| Yes | 11 (11.6) | 4 (6.8) |  | 15 (9.7) |
| RAD21 mutation n(%) |  |  | .483 |  |
| No | 88 (92.6) | 57 (96.6) |  | 145 (94.2) |
| Yes | 7 (7.4) | 2 (3.4) |  | 9 (5.8) |
| RIT1 mutation n(%) |  |  | 1 |  |
| No | 94 (98.9) | 59 (100.0) |  | 153 (99.4) |
| Yes | 1 (1.1) | 0 (0.0) |  | 1 (0.6) |
| RUNX1 mutation n(%) |  |  | .676 |  |
| No | 85 (89.5) | 54 (91.5) |  | 139 (90.3) |
| Yes | 10 (10.5) | 5 (8.5) |  | 15 (9.7) |
| SETBP1 mutation n(%) |  |  | . |  |
| No | 95 (100.0) | 59 (100.0) |  | 154 (100.0) |
| SF3B1 mutation n(%) |  |  | 1 |  |
| No | 93 (97.9) | 58 (98.3) |  | 151 (98.1) |
| Yes | 2 (2.1) | 1 (1.7) |  | 3 (1.9) |
| SH2B3 mutation n(%) |  |  | 1 |  |
| No | 94 (98.9) | 59 (100.0) |  | 153 (99.4) |
| Yes | 1 (1.1) | 0 (0.0) |  | 1 (0.6) |
| SMC1A mutation n(%) |  |  | .086 |  |
| No | 92 (96.8) | 53 (89.8) |  | 145 (94.2) |
| Yes | 3 (3.2) | 6 (10.2) |  | 9 (5.8) |
| SMC3 mutation n(%) |  |  | 1 |  |
| No | 94 (98.9) | 58 (98.3) |  | 152 (98.7) |
| Yes | 1 (1.1) | 1 (1.7) |  | 2 (1.3) |
| SOS1 mutation n(%) |  |  | .524 |  |
| No | 93 (97.9) | 59 (100.0) |  | 152 (98.7) |
| Yes | 2 (2.1) | 0 (0.0) |  | 2 (1.3) |
| SRSF2 mutation n(%) |  |  | .251 |  |
| No | 89 (93.7) | 58 (98.3) |  | 147 (95.5) |
| Yes | 6 (6.3) | 1 (1.7) |  | 7 (4.5) |
| STAG2 mutation n(%) |  |  | .082 |  |
| No | 89 (93.7) | 59 (100.0) |  | 148 (96.1) |
| Yes | 6 (6.3) | 0 (0.0) |  | 6 (3.9) |
| TET2 mutation n(%) |  |  | .756 |  |
| No | 72 (75.8) | 46 (78.0) |  | 118 (76.6) |
| Yes | 23 (24.2) | 13 (22.0) |  | 36 (23.4) |
| TP53 mutation n(%) |  |  | .637 |  |
| No | 93 (97.9) | 57 (96.6) |  | 150 (97.4) |
| Yes | 2 (2.1) | 2 (3.4) |  | 4 (2.6) |
| U2AF1 mutation n(%) |  |  | . |  |
| No | 95 (100.0) | 59 (100.0) |  | 154 (100.0) |
| WT1 mutation n(%) |  |  | .188 |  |
| No | 87 (91.6) | 50 (84.7) |  | 137 (89.0) |
| Yes | 8 (8.4) | 9 (15.3) |  | 17 (11.0) |
| ZRSR2 mutation n(%) |  |  | .157 |  |
| No | 94 (98.9) | 56 (94.9) |  | 150 (97.4) |
| Yes | 1 (1.1) | 3 (5.1) |  | 4 (2.6) |
| FLT3-ITD mutation n(%) |  |  | .352 |  |
| No | 54 (56.8) | 29 (49.2) |  | 83 (53.9) |
| Yes | 41 (43.2) | 30 (50.8) |  | 71 (46.1) |
| FLT3-ITD Ratio>50 n(%) |  |  | .801 |  |
| FLT3-ITD High | 19 (46.3) | 13 (43.3) |  | 32 (45.1) |
| FLT3-ITD Low | 22 (53.7) | 17 (56.7) |  | 39 (54.9) |

**Supplementary Table 3: Clinical characteristics and actionable mutations according to genomic classification.**

|  | **Genomic classification** | |  |  |
| --- | --- | --- | --- | --- |
|  | **NPM1 mutations** | **Mutated chromatin or RNA-splicing genes** | ***P*** | **Total** |
|  | 80 (73.4) | 29 (26.6) |  | 109 (100.0) |
| **Gender - n (%)** |  |  | .479 |  |
| Male | 42 (52.5) | 13 (44.8) |  | 55 (50.5) |
| Female | 38 (47.5) | 16 (55.2) |  | 54 (49.5) |
| **Age - years** |  |  | .954 |  |
| Median | 61.30 | 58.71 |  | 61.03 |
| IQR | [ 49.24; 69.14] | [50.89; 66.65] |  | [49.98; 68.67] |
| **Age - n (%)** |  |  | .697 |  |
| <60 | 38 (47.5) | 15 (51.7) |  | 53 (48.6) |
| >=60 | 42 (52.5) | 14 (48.3) |  | 56 (51.4) |
| **ECOG performance, status - n (%)** |  |  | .001 |  |
| 0-1 | 48 (73.8) | 22 (84.6) |  | 70 (76.9) |
| 2-3-4 | 17 (26.2) | 4 (15.4) |  | 21 (23.1) |
| **Extramedullary involvement - n (%)** |  |  | .503 |  |
| No | 35 (50.0) | 11 (42.3) |  | 46 (47.9) |
| Yes | 35 (50.0) | 15 (57.7) |  | 50 (52.1) |
| **AML status - n (%)** |  |  | <.0001 |  |
| De novo | 74 (92.5) | 18 (62.1) |  | 92 (84.4) |
| Secondary | 6 (7.5) | 11 (37.9) |  | 17 (15.6) |
| **Leukostasis - n (%)** |  |  | .727 |  |
| No | 60 (75.0) | 24 (82.8) |  | 84 (77.1) |
| CNS | 4 (5.0) | 2 (6.9) |  | 6 (5.5) |
| Lung | 12 (15.0) | 3 (10.3) |  | 15 (13.8) |
| CNS and lung | 4 (5.0) | 0 (0.0) |  | 4 (3.7) |
| **Infection at diagnosis – n (%)** |  |  | .127 |  |
| No | 59 (75.6) | 25 (89.3) |  | 84 (79.2) |
| Yes | 19 (24.4) | 3 (10.7) |  | 22 (20.8) |
| **White cell count - x 10^9^/L** |  |  | .293 |  |
| Median | 98.40 | 79.70 |  | 95.25 |
| IQR | [76.95;124.72] | [65.60; 124.00] |  | [72.30; 124.00] |
| **White cell count – x 10^9^/L – n (%)** |  |  | .225 |  |
| <100 | 42 (52.5) | 19 (65.5) |  | 61 (56.0) |
| >=100 | 38 (47.5) | 10 (34.5) |  | 48 (44.0) |
| **Platelet count - x 10^9^/L - n (%)** |  |  | .079 |  |
| <50 | 40 (50.0) | 9 (31.0) |  | 49 (45.0) |
| >=50 | 40 (50.0) | 20 (69.0) |  | 60 (55.0) |
| **ELN 2017 – n (%)** |  |  | .0001 |  |
| Favorable | 49 (61.3) | 0 (0.0) |  | 49 (45.0) |
| Intermediate | 27 (33.8) | 6 (20.7) |  | 33 (30.3) |
| Adverse | 4 (5.0) | 23 (79.3) |  | 27 (24.8) |
| **CD56 - n (%)** |  |  | .398 |  |
| ≤20% | 57 (75.0) | 20 (83.3) |  | 77 (77.0) |
| >20% | 19 (25.0) | 4 (16.7) |  | 23 (23.0) |
| **Creatinine (µM/L) - n (%)** |  |  | 1 |  |
| <=120 | 70 (87.5) | 25 (86.2) |  | 95 (87.2) |
| >120 | 10 (12.5) | 4 (13.8) |  | 14 (12.8) |
| **Bilirubin (µM/L) - n (%)** |  |  | 1 |  |
| <=25 | 75 (96.2) | 28 (96.6) |  | 103 (96.3) |
| >25 | 3 (3.8) | 1 (3.4) |  | 4 (3.7) |
| **Albumin (g/L) - n (%)** |  |  | .072 |  |
| <35 | 38 (48.1) | 8 (28.6) |  | 46 (43.0) |
| >=35 | 41 (51.9) | 20 (71.4) |  | 61 (57.0) |
| **Ferritin (µg/L) - n (%)** |  |  | .190 |  |
| <=1000 | 22 (40.7) | 10 (58.8) |  | 32 (45.1) |
| >1000 | 32 (59.3) | 7 (41.2) |  | 39 (54.9) |
| **LDH - n (%)** |  |  | .409 |  |
| <=1550 | 43 (53.8) | 13 (44.8) |  | 56 (51.4) |
| >1550 | 37 (46.3) | 16 (55.2) |  | 53 (48.6) |
| **Fibrinogen (g/L) - n (%)** |  |  | .435 |  |
| <=1.5 | 5 (6.3) | 3 (10.3) |  | 8 (7.3) |
| >1.5 | 75 (93.8) | 26 (89.7) |  | 101 (92.7) |
| **Hydroxyurea - n (%)** |  |  | .057 |  |
| No | 29 (36.3) | 5 (17.2) |  | 34 (31.2) |
| Yes | 51 (63.8) | 24 (82.8) |  | 75 (68.8) |
| **Admission to intensive care unit - n (%)** |  |  | .086 |  |
| No | 56 (70.0) | 25 (86.2) |  | 81 (74.3) |
| Yes | 24 (30.0) | 4 (13.8) |  | 28 (25.7) |
| **Allo-SCT - n(%)** |  |  | .021 |  |
| No | 62 (77.5) | 16 (55.2) |  | 78 (71.6) |
| Yes | 18 (22.5) | 13 (44.8) |  | 31 (28.4) |
| **Actionable mutations** |  |  |  |  |
| **FLT3 mutation – n (%)** |  |  | .001 |  |
| No | 19 (23.8) | 17 (58.6) |  | 36 (33.0) |
| Yes | 61 (76.3) | 12 (41.4) |  | 73 (67.0) |
| **FLT3-TKD mutation – n (%)** |  |  | .194 |  |
| No | 63 (78.8) | 26 (89.7) |  | 89 (81.7) |
| Yes | 17 (21.3) | 3 (10.3) |  | 20 (18.3) |
| **FLT3-ITD mutation - n (%)** |  |  | .004 |  |
| No | 31 (38.8) | 20 (69.0) |  | 51 (46.8) |
| Yes | 49 (61.3) | 9 (31.0) |  | 58 (53.2) |
| **IDH1 mutation – n (%)** |  |  | .345 |  |
| No | 68 (85.0) | 27 (93.1) |  | 95 (87.2) |
| Yes | 12 (15.0) | 2 (6.9) |  | 14 (12.8) |
| **IDH2 mutation – n (%)** |  |  | .345 |  |
| No | 68 (85.0) | 27 (93.1) |  | 95 (87.2) |
| Yes | 12 (15.0) | 2 (6.9) |  | 14 (12.8) |
| **JAK2 mutation – n (%)** |  |  | .266 |  |
| No | 80 (100.0) | 28 (96.6) |  | 108 (99.1) |
| Yes | 0 (0.0) | 1 (3.4) |  | 1 (0.9) |
| **KIT mutation – n (%)** |  |  | .563 |  |
| No | 77 (96.3) | 29 (100.0) |  | 106 (97.2) |
| Yes | 3 (3.8) | 0 (0.0) |  | 3 (2.8) |
| **TP53 mutation - n (%)** |  |  | 1 |  |
| No | 79 (98.8) | 29 (100.0) |  | 108 (99.1) |
| Yes | 1 (1.3) | 0 (0.0) |  | 1 (0.9) |
| **At least 1 actionable mutation - n (%)** |  |  | <.0001 |  |
| No | 14 (17.5) | 15 (51.7) |  | 29 (26.6) |
| Yes | 66 (82.5) | 14 (48.3) |  | 80 (73.4) |

**Supplementary Table 4: Clinical characteristics and actionable mutations according to ELN 2017 classification.**

|  | **ELN 2017** | | |  |  |
| --- | --- | --- | --- | --- | --- |
|  | **Favorable** | **Intermediate** | **Adverse** | ***P*** | **Total** |
|  | 66 (42.9) | 44 (28.6) | 44 (28.6) |  | 154 (100.0) |
| **Gender - n (%)** |  |  |  | .852 |  |
| Male | 35 (53.0) | 23 (52.3) | 21 (47.7) |  | 79 (51.3) |
| Female | 31 (47.0) | 21 (47.7) | 23 (52.3) |  | 75 (48.7) |
| **Age - years** |  |  |  | .195 |  |
| Median | 56.4 | 61.3 | 62.4 |  | 59.8 |
| IQR | [44.0-66.6] | [52.9-71.3] | [51.6-66.3] |  | [48.7-67.2] |
| **Age - n (%)** |  |  |  | .330 |  |
| <60 | 38 (57.6) | 20 (45.5) | 20 (45.5) |  | 78 (50.6) |
| >=60 | 28 (42.4) | 24 (54.5) | 24 (54.5) |  | 76 (49.4) |
| **ECOG performance, status - n (%)** |  |  |  | .460 |  |
| 0-1 | 38 (69.1) | 26 (78.8) | 25 (65.8 ) |  | 89 (70.6) |
| 2-3-4 | 17 (30.9) | 7 (21.2) | 13 (34.2) |  | 37 (29.4) |
| **Extramedullary involvement - n (%)** |  |  |  | .193 |  |
| No | 30 (49.2) | 17 (50.0) | 13 (32.5) |  | 60 (44.4) |
| Yes | 31 (50.8) | 17 (50.0) | 27 (67.5) |  | 75 (55.6) |
| **AML status - n (%)** |  |  |  | .008 |  |
| De novo | 63 (95.5) | 36 (81.8) | 33 (75.0) |  | 132 (85.7) |
| Secondary | 3 (4.5) | 8 (18.2) | 11 (25.0) |  | 22 (14.3) |
| **Leukostasis - n (%)** |  |  |  | .690 |  |
| No | 49 (74.2) | 31 (70.5) | 34 (77.3) |  | 114 (74.0) |
| CNS | 5 (7.6) | 3 (6.8) | 3 (6.8) |  | 11 (7.1) |
| Lung | 10 (15.2) | 5 (11.4) | 5 (11.4) |  | 20 (13.0) |
| CNS and lung | 2 (3.0) | 5 (11.4) | 2 (4.5) |  | 9 (5.8) |
| **Infection at diagnosis – n (%)** |  |  |  | .505 |  |
| No | 51 (78.5) | 31 (73.8) | 37 (84.1) |  | 119 (78.8) |
| Yes | 14 (21.5) | 11 (26.2) | 7 (15.9) |  | 32 (21.2) |
| **White cell count - x 10^9^/L** |  |  |  | .708 |  |
| Median | 108.0 | 91.8 | 94.6 |  | 97.6 |
| IQR | [80.0-126.4] | [68.9-146.1] | [69.1-157.8] |  | [70.8-137.0] |
| **White cell count – x 10^9^/L – n (%)** |  |  |  | .185 |  |
| <100 | 29 (43.9) | 27 (61.4) | 24 (54.5) |  | 80 (51.9) |
| >=100 | 37 (56.1) | 17 (38.6) | 20 (45.5) |  | 74 (48.1) |
| **Platelet count - x 10^9^/L - n (%)** |  |  |  | .309 |  |
| <50 | 33 (50.0) | 22 (50.0) | 16 (36.4) |  | 71 (46.1) |
| >=50 | 33 (50.0) | 22 (50.0) | 28 (63.6) |  | 83 (53.9) |
| **CD56 - n (%)** |  |  |  | .627 |  |
| ≤20% | 48 (77.4) | 35 (81.4) | 29 (72.5) |  | 112 (77.2) |
| >20% | 14 (22.6) | 8 (18.6) | 11 (27.5) |  | 33 (22.8) |
| **Creatinine (µM/L) - n (%)** |  |  |  | .003 |  |
| <=120 | 59 (89.4) | 39 (88.6) | 29 (65.9) |  | 127 (82.5) |
| >120 | 7 (10.6) | 5 (11.4) | 15 (34.1) |  | 27 (17.5) |
| **Bilirubin (µM/L) - n (%)** |  |  |  | .625 |  |
| <=25 | 60 (93.8) | 43 (97.7) | 42 (95.5) |  | 145 (95.4) |
| >25 | 4 (6.3) | 1 (2.3) | 2 (4.5) |  | 7 (4.6) |
| **Albumin (g/L) - n (%)** |  |  |  | .034 |  |
| <35 | 26 (40.0) | 26 (59.1) | 14 (32.6) |  | 66 (43.4) |
| >=35 | 39 (60.0) | 18 (40.9) | 29 (67.4) |  | 86 (56.6) |
| **Ferritin (µg/L) - n (%)** |  |  |  | .557 |  |
| <=1000 | 20 (47.6) | 11 (40.7) | 16 (55.2) |  | 47 (48.0) |
| >1000 | 22 (52.4) | 16 (59.3) | 13 (44.8) |  | 51 (52.0) |
| **LDH - n (%)** |  |  |  | .348 |  |
| <=1550 | 38 (57.6) | 20 (45.5) | 18 (40.9) |  | 76 (49.4) |
| >1550 | 28 (42.4) | 24 (54.5) | 26 (59.1) |  | 78 (50.6) |
| **Fibrinogen (g/L) - n (%)** |  |  |  | .220 |  |
| <=1.5 | 3 (4.5) | 5 (11.4) | 6 (13.6) |  | 14 (9.1) |
| >1.5 | 63 (95.5) | 39 (88.6) | 38 (86.4) |  | 140 (90.9) |
| **Hydroxyurea - n (%)** |  |  |  | .099 |  |
| No | 27 (40.9) | 12 (27.3) | 10 (22.7) |  | 49 (31.8) |
| Yes | 39 (59.1) | 32 (72.7) | 34 (77.3) |  | 105 (68.2) |
| **Admission to intensive care unit - n (%)** |  |  |  | .960 |  |
| No | 48 (72.7) | 31 (70.5) | 32 (72.7) |  | 111 (72.1) |
| Yes | 18 (27.3) | 13 (29.5) | 12 (27.3) |  | 43 (27.9) |
| **Allo-SCT - n(%)** |  |  |  | .046 |  |
| No | 53 (80.3) | 33 (75.0) | 26 (59.1) |  | 112 (72.7) |
| Yes | 13 (19.7) | 11 (25.0) | 18 (40.9) |  | 42 (27.3) |
| **Actionable mutations** |  |  |  |  |  |
| **FLT3 mutation – n (%)** |  |  |  | .116 |  |
| No | 23 (34.8) | 13 (29.5) | 22 (50.0) |  | 58 (37.7) |
| Yes | 43 (65.2) | 31 (70.5) | 22 (50.0) |  | 96 (62.3) |
| **FLT3-TKD mutation – n (%)** |  |  |  | .004 |  |
| No | 45 (68.2) | 40 (90.9) | 39 (88.6) |  | 124 (80.5) |
| Yes | 21 (31.8) | 4 (9.1) | 5 (11.4) |  | 30 (19.5) |
| **FLT3-ITD mutation - n (%)** |  |  |  | .022 |  |
| No | 40 (60.6) | 16 (36.4) | 27 (61.4) |  | 83 (53.9) |
| Yes | 26 (39.4) | 28 (63.6) | 17 (38.6) |  | 71 (46.1) |
| **IDH1 mutation – n (%)** |  |  |  | .175 |  |
| No | 58 (87.9) | 39 (88.6) | 43 (97.7) |  | 140 (90.9) |
| Yes | 8 (12.1) | 5 (11.4) | 1 (2.3) |  | 14 (9.1) |
| **IDH2 mutation – n (%)** |  |  |  | .881 |  |
| No | 58 (87.9) | 40 (90.9) | 39 (88.6) |  | 137 (89.0) |
| Yes | 8 (12.1) | 4 (9.1) | 5 (11.4) |  | 17 (11.0) |
| **JAK2 mutation – n (%)** |  |  |  | .284 |  |
| No | 66 (100.0) | 44 (100.0) | 43 (97.7) |  | 153 (99.4) |
| Yes | 0 (0.0) | 0 (0.0) | 1 (2.3) |  | 1 (0.6) |
| **KIT mutation – n (%)** |  |  |  | .053 |  |
| No | 58 (87.9) | 41 (93.2) | 44 (100.0) |  | 143 (92.9) |
| Yes | 8 (12.1) | 3 (6.8) | 0 (0.0) |  | 11 (7.1) |
| **TP53 mutation - n (%)** |  |  |  | .006 |  |
| No | 66 (100.0) | 44 (100.0) | 40 (90.9) |  | 150 (97.4) |
| Yes | 0 (0.0) | 0 (0.0) | 4 (9.1) |  | 4 (2.6) |
| **At least 1 actionable mutation - n (%)** |  |  |  | .224 |  |
| No | 15 (22.7) | 10 (22.7) | 16 (36.4) |  | 41 (26.6) |
| Yes | 51 (77.3) | 34 (77.3) | 28 (63.6) |  | 113 (73.4) |

**Supplementary Table 5: Multivariate Analysis for Overall Survival including Individual Gene Mutations selected by LASSO.**

|  | **Numbers** | **Events** | **HR** | **95% CI** | ***P* value** |
| --- | --- | --- | --- | --- | --- |
| **Dexamethasone***  No  Yes | 95  59 | 70  26 | 1  0.34 | 0.20-0.57 | <0.001 |
| **Individual genes (LASSO)***  CBFB-MYH11  No  Yes  CEBPA  No  Yes  DNMT3A  No  Yes  NPM1  No  Yes  RUNX1  No  Yes | 141  13  141  13  101  53  72  82  139  15 | 94  2  89  7  55  41  45  51  88  8 | 1  0.10  1  0.22  1  1.76  1  0.33  1  0.40 | 0.02-0.43  0.09-0.54  1.02-3.03  0.19-0.58  0.18-0.92 | 0.002  0.001  0.043  <0.001  0.030 |
| **AML status**  De novo  Secondary | 132  22 | 77  19 | 1  2.33 | 1.26-4.30 | 0.007 |
| **Albumin - g/L**  < 35  ≥ 35 | 66  86 | 43  51 | 1  0.46 | 0.29-0.73 | 0.001 |
| **LDH – IU/L**  ≤1550  >1550 | 76  78 | 38  58 | 1  1.64 | 1.02-2.64 | 0.043 |
| **Fibrinogen – g/L**  ≤ 1.5  > 1.5 | 14  140 | 11  85 | 1  0.38 | 0.19-0.78 | 0.008 |
| **Hydroxyurea**  No  Yes | 49  105 | 35  61 | 1  0.59 | 0.37-0.94 | 0.012 |
| **Admission to intensive care unit****  No  Yes | 111  43 | 68  28 | 1  4.60 | 2.49-8.50 | <0.001 |
| **Allogeneic stem cell transplantation*****  No  Yes | 112  42 | 76  20 | 1  0.39 | 0.21-0.74 | 0.004 |

* An interaction between dexamethasone and the presence of gene mutations selected by LASSO (least absolute shrinkage and selection operator) was tested but was not significant, indicating that the effect of dexamethasone does not differ significantly depending on the presence or absence of these mutations. ** during the first three months following chemotherapy. *** Allogeneic stem-cell transplantation was evaluated as a time-dependent covariate. HR, hazard ratio; CI, confidence interval.

**Supplementary Table 6: RUNX1^mut^ AML: Type of Mutations and Outcome.**

| **Patients (n=15)** | **RUNX1 mutations** | **VAF** | **Co-mutations** | **DEX (y/no)** | **CR (y/no)** | **Alive (y/no)** | **RUNX1 mutations** |
| --- | --- | --- | --- | --- | --- | --- | --- |
| 2007355 | Missense | 47% | ASXL1, EZH2, RAD21, STAG2, TET2 | no | No | y | NM_001754.4(RUNX1):c.473T>C, NM_001754.4(RUNX1):p.Phe158Ser |
| 2009443 | Nonsense | 42% | NPM1, PTPN11, WT1 | no | y | no | NM_001754.4(RUNX1):c.958C>T, NM_001754.4(RUNX1):p.Arg320*; |
| 20111053 | Missense | 45% | ATM, WT1, t(9;22) | no | y | no | NM_001754.4(RUNX1):c.602G>A, NM_001754.4(RUNX1):p.Arg201Gln |
| 2010775 | Missense | 22% | BCORL1, FLT3-TKD | no | y | no | NM_001754.4(RUNX1):c.1270T>G, NM_001754.4(RUNX1):p.Ser424Ala |
| 20141652 | Missense | 45% | ASXL1, FLT3 | no | y | y | NM_001754.4(RUNX1):c.293T>C, NM_001754.4(RUNX1):p.Leu98Pro |
| 2006289 | Missense | 42% | FLT3-ITD | no | y | y | NM_001754.4(RUNX1):c.602G>A, NM_001754.4(RUNX1):p.Arg201Gln |
| 20131389 | Frameshift | 85% | ASXL1, DNMT3A, FLT3-ITD | no | Y | y | NM_001754.4(RUNX1):c.492_493insA, NM_001754.4(RUNX1):p.Gly165fs*48 |
| 2006462 | Frameshift | 43% | BCOR, DNMT3A, IDH1 | no | Y | y | NM_001754.4(RUNX1):c.1182_1185dupCCCG, NM_001754.4(RUNX1):Phe396Profs*205; |
| 2009437 | Frameshift, Missense, Missense | 1%, 74%, 4% | ASXL1, NRAS | no | y | y | NM_001754.4(RUNX1):c.1090_1103delATCGGCATCGGCAT,  NM_001754.4(RUNX1):p.Ile364Valfs*231; NM_001754.4(RUNX1):c.593A>G, NM_001754.4(RUNX1):p.Asp198Gly; NM_001754.4(RUNX1):c.274A>G, NM_001754.4(RUNX1):p.Thr92Ala |
| 2008494 | Frameshift | 36% | ASXL1, FLT3-ITD, SRSF2 | no | y | y | NM_001754.4(RUNX1):c.968_976delinsGGGCATAC, NM_001754.4(RUNX1):p.Thr323Argfs*5 |
| 20151608 | Frameshift | 43% | FLT3-ITD, WT1 | y | no | y | NM_001754.4(RUNX1):c.952delT, NM_001754.4(RUNX1):p.Ser318Profs*10 |
| 20151615 | Missense | 37% | FLT3-ITD (x2), WT1 (x2) | y | y | no | NM_001754.4(RUNX1):c.601C>G, NM_001754.4(RUNX1):p.Arg201Gly |
| 2010785 | Frameshift | 97% | DNMT3A, IDH2, KDM6A, MGA, SF3B1 | y | y | no | NM_001754.4(RUNX1):c.820delC, NM_001754.4(RUNX1):p.Gln274Asnfs*37 |
| 2010815 | Frameshift | 42% | FLT3-ITD, JAK2 | y | y | no | NM_001754.4(RUNX1):c.423_424insTGGCGCCTCCCGGCCGNM_001754.4(RUNX1):p.Ala142Trpfs*7 |
| 20151635 | Nonsense | 63% | t(9;22) | y | y | no | NM_001754.4(RUNX1):c.601C>T, NM_001754.4(RUNX1):p.Arg201* |

VAF, variant allelic frequency; DEX, dexamethasone, CR, complete response.
